# Dataset from transcriptome profiling of *Musa* resistant and susceptible cultivars in response to *Fusarium oxysporum* f.sp. *cubense* race1 and TR4 challenges using Illumina NovaSeq

**DOI:** 10.1016/j.dib.2023.109803

**Published:** 2023-11-13

**Authors:** C. Anuradha, A. Chandrasekar, S. Backiyarani, R. Thangavelu, S. Uma, R. Selvarajan

**Affiliations:** ICAR-National Research Centre for Banana, Thogamalai Road, Thayanur Post, Tiruchirappalli 620102, Tamil Nadu, India

**Keywords:** Banana, *Fusarium oxysporum* f.sp. *cubense*, Transcriptome, Gene-expression, Annotation, Illumina sequencing

## Abstract

In this investigation, the study focused on the RNAseq data generated in response to *Fusarium oxysporum* f.sp. *cubense* (Foc) race1 (Cavendish infecting strain VCG 0124), targeting both resistant (cv. Rose, AA) and susceptible cultivars (Namarai, AA), and Tropical Race 4 (TR4, strain VCG 01213/16), involving resistant (cv. Rose, AA) and susceptible cultivars (Matti, AA). The respective contrasting cultivars were independently challenged with Foc race1 and TR4, and the root and corm samples were collected in two replications at varying time intervals [0th (control), 2nd, 4th, 6th, and 8th days] in duplicates. The RNA samples underwent stringent quality checks, with all 80 samples meeting the primary parameters, including a satisfactory RNA integrity number (>7). Subsequent library preparation and secondary quality control steps were executed successfully for all samples, paving the way for the sequencing phase. Sequencing generated an extensive amount of data, yielding a range of 10 to 31 million paired-end raw reads per sample, resulting in a cumulative raw data size of 11–50 GB. These raw reads were aligned against the reference genome of *Musa acuminata* ssp. *malaccensi*s version 2 (DH Pahang), as well as the pathogen genomes of Foc race 1 and Foc TR4, using the HISAT2 alignment tool. The focal point of this study was the investigation of differential gene expression patterns of *Musa* spp. upon Foc infection. In Foc race1 resistant and susceptible root samples across the designated day intervals, a significant number of genes displayed up-regulation (ranging from 1 to 228) and down-regulation (ranging from 1 to 274). In corm samples, the up-regulated genes ranged from 1 to 149, while down-regulated genes spanned from 3 to 845. For Foc TR4 resistant and susceptible root samples, the expression profiles exhibited a notable up-regulation of genes (ranging from 31 to 964), along with a down-regulation range of 316–1315. In corm samples, up-regulated genes ranged from 57 to 929, while down-regulated genes were observed in the range of 40–936. In addition to the primary analysis, a comprehensive secondary analysis was conducted, including Gene Ontology (GO), euKaryotic Orthologous Groups (KOG) classification, Kyoto Encyclopedia of Genes and Genomes (KEGG) pathway analysis, and investigations into Simple Sequence Repeats (SSRs), Single Nucleotide Polymorphisms (SNPs), and microRNA (miRNA). The complete dataset was carefully curated and housed at ICAR-NRCB, Trichy, ensuring its accuracy and accessibility for the duration of the study. Further, the raw transcriptome read datasets have been successfully submitted to the National Center for Biotechnology Information - Sequence Read Archive (NCBI-SRA) database, ensuring the accessibility and reproducibility of this valuable dataset for further research endeavors

Specification Table

**Table:** 

Subject	Plant Science: Host pathogen interaction
Specific subject area	Transcriptome and global gene expression of resistant and susceptible banana roots and corms challenged with Foc race 1 (Cavendish infecting strain) and TR4.
Data format	Raw (FASTQ) sequences, Analyzed, Filtered
Type of data	Table, text file, figure
How the data were acquired	Illumina NovaSeq 6000 System, Fastp, Subread, Hisat2, edgeR, Freebayes, Misa, Mirdeep, CPC webserver, EggNOG, KAAS, SignalP, EffectorP
Data collection	The RNAseq read assembly was carried out utilizing Illumina data and employing the Burrows-Wheeler Transform (BWT) and Hierarchical indexing for spliced alignment of transcripts (HISAT2) algorithm for accurately aligning sequences based on the specific traits of the reference genome *M.ac. malaccensis* 2.0. Across various conditions, the assembly yielded an average of approximately 414,789 and 340,769 contigs from the corm and root tissues of resistant cultivars, 407,178 and 344,629 contigs from the corm and root tissues of susceptible cultivars following Foc race1 infection, and 346,781 and 418,680 contigs from the corm and root tissues of resistant cultivars, as well as 401,097 and 429,383 contigs from the corm and root tissues of susceptible cultivars upon TR4 challenge, respectively. Furthermore, comprehensive analyses were performed on the transcriptome assembly, including gene prediction, functional annotation, and exploration of GO, KEGG pathways, KOG, as well as various pathways. Secondary analyses encompassing SSR, SNP, and miRNA were also executed as part of the analysis process.
Data source location	Institution: ICAR-National Research Centre for Banana City/Town/Region: Tiruchirappalli, Tamil Nadu Country: India Latitude and longitude (and GPS coordinates) for collected samples/data: (11.500, 74.500) 10° 48’ 55.8” N, 78° 41’ 47.436” E.
Data accessibility	Repository name: NCBI SRA Database Accession to cite for these SRA data: Foc race1: PRJNA995936. Foc TR4: PRJNA1000769 Temporary Submission ID: Foc race1: SUB13675851. Foc TR4: SUB13692569 Release date: 2024-01-31 Bio Sample Accession No.: Foc race1: SAMN36510589 - SAMN36510628 Foc TR4: SAMN36780136 - SAMN36780175 Direct URL to data: Foc race1: https://www.ncbi.nlm.nih.gov/sra/PRJNA995936 Foc TR4: https://www.ncbi.nlm.nih.gov/sra/PRJNA1000769
Related research article	• Anuradha C, Chandrasekar A, Backiyarani S, Thangavelu R and Uma S (2022b). Genome-wide identification, characterization, and evolutionary analysis of NBS genes and their association with disease resistance in *Musa* spp. Functional & Integrative Genomics, 23:7

## 1 Value of the Data

• This information uncovers a range of downstream analyses, including assembly, annotation, differential expression, pathway investigation, and exploration of interactions between *Musa* spp. and the respective Foc race 1 and TR4.
• By examining the transcriptome profiles and annotations of Fusarium wilt resistant and susceptible cultivars that are challenged with Foc race1 and TR4 independently, we can gain insights into the intricate molecular mechanisms that drive defense pathways in banana.
• The outcomes aid in pinpointing potential genes associated with plant resistance within the non-model organism, banana. Furthermore, they enhance our existing comprehension of interactions between hosts and pathogens.
• The transcriptome sequences will function as essential references and valuable reservoirs for investigating genes linked to resistance and defense, which hold significant roles in *Musa* cultivars afflicted by Foc race1 and TR4 infections.
• Repurposing the data facilitates the creation of markers for Marker-Assisted Selection, enabling breeders to precisely target and enhance desired traits, particularly in relation to Fusarium wilt resistance.
• Employing the data in Genome-Wide Association Studies aids in the identification of candidate genes responsible for controlling plant immunity.
• Utilizing the data empowers breeders to develop new cultivars with heightened resistance to Fusarium wilt. The information obtained from these data will provide a baseline understanding of the genes of practical importance in a resistance breeding programme.

## 2 Objective of the Study

The primary objective of this study is to comparing the infection responses of diploid-resistant and susceptible banana cultivars to Foc race 1 (the Cavendish-infecting strain) and TR4. To achieve this goal, a comprehensive analysis of the global transcriptome responses has been conducted in three banana cultivars: cv Rose (AA, resistant to both races), Namarai (AA, susceptible to Foc race 1), and Matti (AA, susceptible to Foc TR4). The study aims to identify differentially expressed genes (DEGs) and elucidate the distinct defense responses triggered by these two Foc races.

## 3 Data Description

**Fig. 1:**
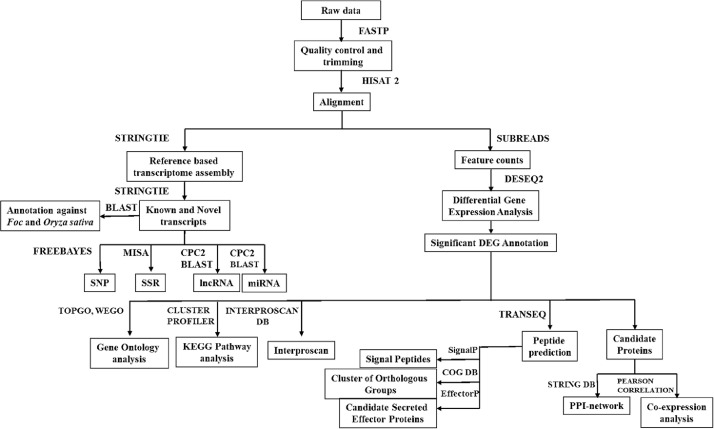
Workflow illustrating the *Musa* transcriptome analysis challenged by Foc race1 and TR4.

Detailed statistical information and pertinent data concerning transcripts and unigenes within the context of Foc race1 and TR4-challenged *Musa* transcriptome profiles have been made available in the Supplementary data 1a, 1b and 2a, 2b (https://data.mendeley.com/datasets/pnnhtxpd23/1). The results revealed an average count of 342,699 and 424,031 unigenes obtained from root samples, while corm samples yielded 410,984 and 373,939 unigenes in the context of Foc race1 and TR4 challenged *Musa* cultivars, respectively. Remarkably, these unigenes displayed an average length of around 1,120 base pairs. The comprehensive annotation process utilized a range of databases, notably KOG, NR, KEGG, InterPro, GO, and Pfam, as outlined in Table 3. The entire workflow detailing the transcriptome analysis of *Musa* samples is visually depicted in Fig. 1. To enhance data accessibility, the raw reads were deposited into the NCBI-SRA database under the following temporary bio sample accession numbers: Foc race1 samples are under SAMN36510589 - SAMN36510628, and Foc TR4 samples are denoted by SAMN36780136 - SAMN36780175. The transcriptome analysis and annotation files, encompassing predicted SSRs, SNPs, KOG, NR, KEGG pathways, and plant transcription factors, have been securely archived on an in-house server.

Pipeline Flowchart

### 3.1 *Musa* Transcriptome reads sequence statistics

Supplementary data 1a and 1b

RNAseq read statistics of Foc race1 and TR4 challenged *Musa* resistant and susceptible cultivars.

Supplementary data 2a and 2b

Read mapping distribution statistics with reference genome.

Supplementary data 4a-4d

RNA-seq raw read counts to genes in the Foc race1 and TR4 challenged corm, root samples (before normalization) https://data.mendeley.com/datasets/g3nwfxy7tx/1.

## 4 Experimental Design, Materials and Methods

### 4.1 Sample collection

The primary focus of the study was centered around the RNAseq data obtained as a result of the interaction between *Musa* species and two distinct strains of Foc race1 (Cavendish infecting strain VCG 0124) [1,2], and Tropical Race 4 (TR4, strain VCG 01213/16) [3]. The investigation targeted both resistant and susceptible cultivars of *Musa*, with specific cultivars being cv Rose (resistant, AA, Accession no. 0638) and Namarai (susceptible, AA, Accession no. 0185) for the Foc race1 strain, and cv Rose (resistant, AA) and Matti (susceptible, AA, Accession no. 0182) for the TR4 strain [4]. The chosen cultivars were subjected to inoculation with Foc race1 and TR4, after which root and corm samples were collected at various day intervals [0th (control), 2nd, 4th, 6th, and 8th days], each with duplicate samples.

### 4.2 Total RNA extraction

Total RNA was isolated from root and corm samples using the RNA extraction kit (Sigma-Aldrich, USA). Total RNA was analyzed by agarose gel electrophoresis for size and integrity. The quantification of total RNA was done with a Nanodrop 2000 (Thermo Scientific™ NanoDrop™ 2000/2000c spectrophotometers). Consequently, the integrity of RNA used for library preparations was checked with a value of >7 using Bioanalyzer (Agilent, USA). The quality control (QC) passed RNA samples were then processed for library preparation.

### 4.3 Library preparation

DNA-free RNA was used for cDNA synthesis and amplification employing the NEBNext® Single Cell for cDNA Synthesis & Amplification Module (E6421), New England Biolabs, Massachusetts, USA. Following cDNA synthesis, the resulting product was purified using Pronexbeads (NG103B) (Promega, Madison, USA). The length of the cDNA library was evaluated using the Agilent TapeStation instrument (Agilent Technologies). Subsequently, the prepared library was subjected to sequencing using the Illumina Novaseq 6000 platform (Illumina, USA).

### 4.4 Transcriptome sequencing and assembly

Raw reads obtained from sequencing were processed to obtain high-quality reads. Moreover, all reads were trimmed by using the Trimmomatic 0.35 tool [5] to remove low-quality reads and any adapter sequences if present. The sequence quality was accessed using fastp tool (FASTQ data pre-processing tool) with default settings [6]. The algorithm has functions to check the quality control, trimming of adapters, filtering by quality, and read pruning. The resultant high-quality reads of each sample were used for mapping on *Musa acuminata* DH-Pahang v2 on banana genome hub (https://banana-genome-hub.southgreen.fr/download) [7,^8^,9] by BWT and HISAT2 [10,11]. The transcriptome assembly pipeline is illustrated in Fig. 1 [12,13].

### 4.5 Functional annotation

**Fig. 2:**
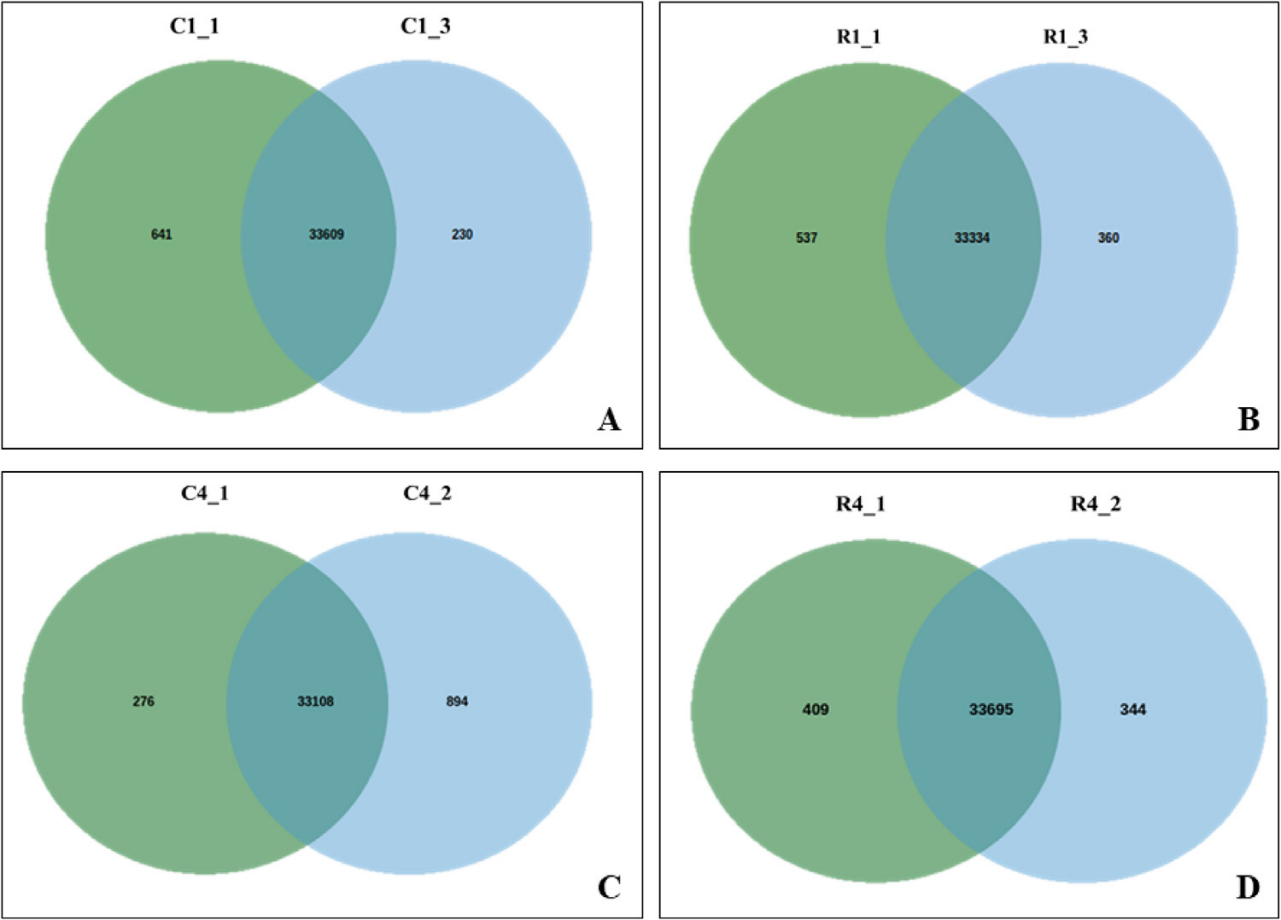
Venn diagrams of commonly expressed genes upon Foc race1 and TR4 infection in root and corm samples of resistant and susceptible cultivars. A) Foc Race1 Corm (Res vs Sus) B) Foc race1 Root (Res vs Sus) C) Foc TR4 Corm (Res vs Sus) D) Foc TR4 Root (Res vs Sus) based on absolute normalized threshold values > 1 per sample.

**Fig. 3:**
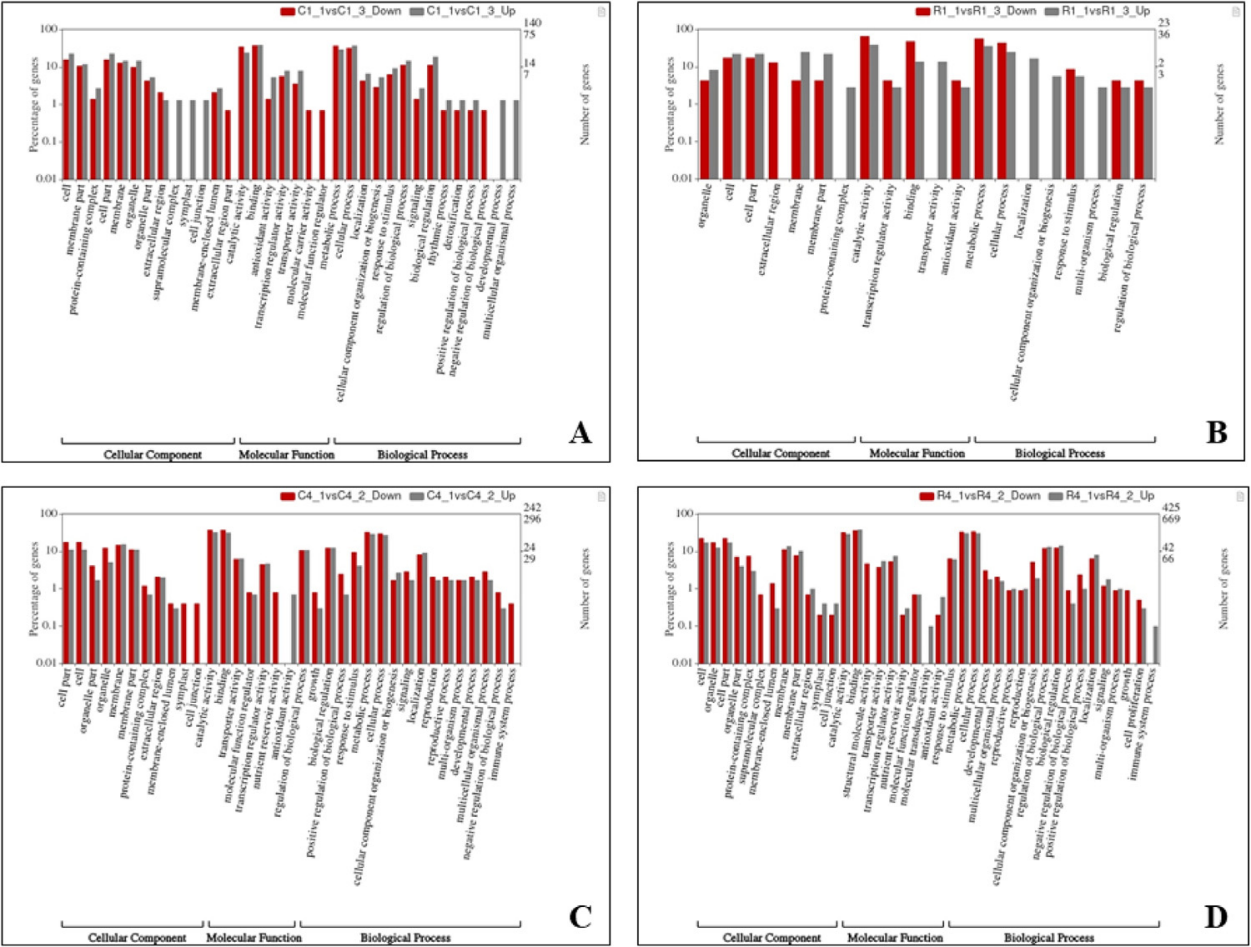
Gene enrichment chart for up and down regulated genes of A) Foc race1 (Corm Sample) Resistant vs Susceptible B) Foc Race1 (Root Sample) Resistant vs Susceptible C) Foc TR4 (Corm Sample) Resistant vs Susceptible D) Foc TR4 (Root Sample) Resistant vs Susceptible.

We utilized the DESeq2 package [14], which is specifically developed for the normalization, visualization, and assessment of differential gene expression (DGE) in datasets with high-dimensional count data. For our comparative analysis between the resistant and susceptible conditions in relation to the control, we employed defined criteria to identify up-regulated and down-regulated genes. Genes were considered up-regulated if their log2 fold change (log2FC) was greater than or equal to 2 and their adjusted *p*-value (padj) was below 0.05. Conversely, genes were deemed down-regulated if their log2 fold change was less than or equal to -2 and their adjusted *p*-value was below 0.05. These criteria enabled us to pinpoint significant changes in gene expression associated with resistance and susceptibility. Following infection with Foc race1, we detected an average of 47 and 34 up-regulated genes, as well as 92 and 133 down-regulated genes, in samples taken from the roots and corms of resistant and susceptible cultivars, respectively. These samples were collected at multiple day intervals [0th (Control), 2nd, 4th, 6th, and 8th]. Similarly, in response to TR4 infection, we observed an average of 564 and 381 up-regulated genes, along with 730 and 477 down-regulated genes, in the roots and corms of resistant and susceptible cultivars, respectively (**Supplementary data 3a-3h**) (https://data.mendeley.com/datasets/pnnhtxpd23/1). The commonly expressed genes upon Foc race1 and TR4 infection in root and corm samples of resistant and susceptible cultivars are provide in Fig. 2. The assembled contigs, which encompassed full-length sequences, underwent annotation through similarity searches against the non-redundant (NR) databases [15], employing an e-value threshold of 1e^−5^. Functional annotation was conducted utilizing both the KOG [16] and GO [17] databases. For KEGG pathway analysis [18], the parameters utilized were as follows: species ko and an E-value cutoff of 1e−5, which facilitated the comparison of annotated transcripts. Furthermore, to assess the distribution of differentially expressed genes (DEGs) across various pathways, we employed the WEGO tool [19] to compute statistical GO enrichment (Fig. 3). The classification of contigs was accomplished using the Pfam database [20]. This comprehensive annotation approach provided valuable insights into the functional characteristics and potential roles of the identified contigs and genes (Tables 1, 2a and 2b).

**Table 1:** Transcriptome summary statistics in average.

Name of the category	Foc race1		Foc TR4	
	Root	Corm	Root	Corm
Total reads	1,64,825,720	1,71,409,102	1,54,292,790	1,59,365,989
Total bases	26,528,055,698	27,626,162,864	24,853,042,909	25,796,248,696
Mean read length replicate 1	161	161	161	161
Mean read length replicate 2	161	161	161	161
Raw data in GB	27	28	25	26
Total number of transcripts	3,42,699	4,10,984	4,24,032	3,73,939
Total number of novel transcripts	3,89,000	4,74,485	4,84,154	4,19,985
Up-regulated genes (Overall)	5476	3118	3973	3532
Down-regulated genes (Overall)	5467	4196	4489	5097
Neutral genes (Overall)	23,288	26,917	25,769	25,602
Up-regulated genes (Significant)	47	34	564	381
Down-regulated genes (Significant)	92	133	730	477
Neutral genes (Significant)	34,134	34,337	33,472	33,640
Total number of identified SSRS	96,751	1,18,524	1,21,300	1,07,262
KOG Analysis (Res vs Sus)	26,536	29,425	27,116	25,412
Gene Ontology (GO Terms) (Res vs Sus) genes	69,375	69,523	69,592	69,409
Gene Ontology (GO Terms) (Res vs Sus)	2308	2308	2308	2308
Pathways	4953	3818	3926	3873
KEGG enrichment analysis (Res vs Sus)	34	15	30	12

**Table 2a:** Total number of contigs from the transcriptomes of *Musa* plants, specifically the corm and root tissues of both resistant and susceptible cultivars, upon challenge with Foc race1 and TR4.

Foc race1				Foc TR4			
Sample C1 (Corm)	Contigs	Sample R1 (Root)	Contigs	Sample C4 (Corm)	Contigs	Sample R4 (Root)	Contigs
C1_1_0	4,16,522	R1_1_0	3,53,391	C4_1_0	4,55,430	R4_1_0	3,61,722
C1_1_2	4,33,593	R1_1_2	2,59,212	C4_1_2	3,60,367	R4_1_2	4,76,196
C1_1_4	3,85,355	R1_1_4	2,16,842	C4_1_4	3,22,609	R4_1_4	4,07,393
C1_1_6	4,37,350	R1_1_6	4,37,366	C4_1_6	2,85,061	R4_1_6	3,97,645
C1_1_8	4,01,128	R1_1_8	4,37,037	C4_1_8	3,10,438	R4_1_8	4,50,447
C1_3_0	4,15,545	R1_3_0	2,94,594	C4_2_0	4,56,900	R4_2_0	3,98,416
C1_3_2	3,83,234	R1_3_2	2,24,116	C4_2_2	4,19,234	R4_2_2	4,09,106
C1_3_4	4,44,702	R1_3_4	4,47,306	C4_2_4	4,44,636	R4_2_4	4,35,362
C1_3_6	3,91,746	R1_3_6	4,19,342	C4_2_6	2,85,399	R4_2_6	4,64,295
C1_3_8	400,667	R1_3_8	3,37,790	C4_2_8	3,99,319	R4_2_8	4,39,738

**Table 2b:** Total number of peptides from the transcriptomes of *Musa* plants, specifically the corm and root tissues of both resistant and susceptible cultivars, upon challenge with Foc race1 and TR4.

Foc race1				Foc TR4			
Sample C1 (Corm)	Peptides	Sample R1 (Root)	Peptides	Sample C4 (Corm)	Peptides	Sample R4 (Root)	Peptides
C1_1_0	4,16,522	R1_1_0	3,40,526	C4_1_0	4,55,430	R4_1_0	3,61,722
C1_1_2	4,33,593	R1_1_2	2,59,212	C4_1_2	3,60,367	R4_1_2	4,76,196
C1_1_4	3,85,355	R1_1_4	2,16,307	C4_1_4	3,14,858	R4_1_4	4,07,393
C1_1_6	4,37,350	R1_1_6	4,37,366	C4_1_6	2,85,061	R4_1_6	3,97,645
C1_1_8	3,98,901	R1_1_8	4,37,020	C4_1_8	3,10,438	R4_1_8	4,50,447
C1_3_0	4,15,545	R1_3_0	2,94,594	C4_2_0	4,56,900	R4_2_0	3,98,416
C1_3_2	3,83,234	R1_3_2	2,24,116	C4_2_2	4,19,234	R4_2_2	4,09,106
C1_3_4	4,44,702	R1_3_4	447,306	C4_2_4	4,44,636	R4_2_4	4,35,362
C1_3_6	3,91,746	R1_3_6	4,17,811	C4_2_6	1,51,246	R4_2_6	4,64,295
C1_3_8	4,00,667	R1_3_8	3,37,790	C4_2_8	3,99,319	R4_2_8	4,39,738

**Table 3:** Statistics of annotation and analysis.

Category	Foc race 1		Foc TR4	
	Root	Corm	Root	Corm
Total KOG annotation	26,536	29,425	27,116	25,412
Total annotation	44,812	36,244	44,860	45,073
No. of filtered pathways	36	36	36	36
Total number of identified SSRs	96,751	1,18,524	1,21,300	1,07,262
Total number of identified SNPs	4,35,7503	4,78,5167	4,72,5111	4,94,6309
Total Pfam domains	3315	3447	3521	3490
Total KAAS - KEGG pathway annotation	406	409	410	407
Differential gene expression	34,231	34,480	34,492	34,279
Transcription factors	2364	2360	2360	2348

## Ethics Statement

The current work meets the ethical requirements for publication in Data in Brief and does not involve human subjects, animal experiments, or any data collected from social media platforms

## CRediT authorship contribution statement

**C. Anuradha:** Project administration, Conceptualization, Methodology, Data curation, Visualization, Formal analysis, Writing – original draft, Writing – review & editing, Supervision, Funding acquisition. **A. Chandrasekar:** Methodology, Writing – original draft. **S. Backiyarani:** Resources. **R. Thangavelu:** Resources. **S. Uma:** Validation. **R. Selvarajan:** Validation.

## Data Availability

Transcriptome analysis of Foc TR4 challenged Musa spp. (Original data) (NCBI SRA) Transcriptome analysis of Foc race1 challenged Musa spp. (Original data) (NCBI SRA) Dataset from transcriptome profiling of Musa resistant and susceptible cultivars in response to Fusarium oxysporum f.sp. cubense race1 and TR4 challenges using Illumina NovaSeq (Original data) (Mendeley Data) RNA-seq raw read counts to genes in the Foc race1 and TR4 challenged corm, root samples (before normalization) (Original data) (Mendeley Data)
